# Supplementary material for: Viral oncogene EBNALP regulates YY1 DNA binding and alters host 3D genome organization
Source: EMBO Rep. 2025 Jan 2;26(3):810–35. doi: 10.1038/s44319-024-00357-6 (PMC11811279; doi:10.1038/s44319-024-00357-6)
Supplement: Supplementary file 1 — Appendix [file 44319_2024_357_MOESM1_ESM.pdf]

Appendix

Appendix Figure S1 Page 2

Appendix Figure S2 Page 3

Appendix Figure S1

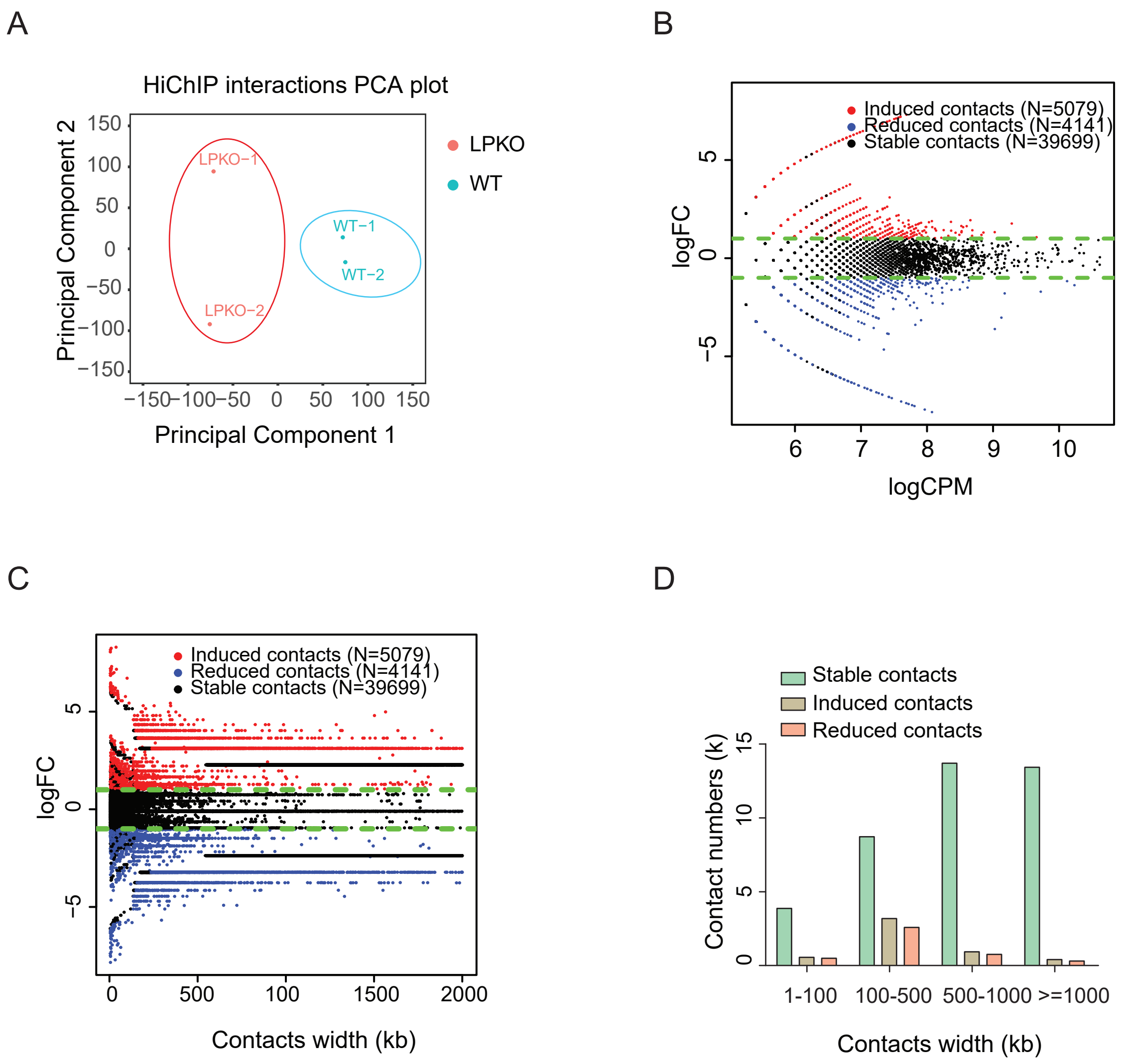

Appendix Figure S1. (A) PCA analysis of H3K27ac HiChIP two replicates from WT and LPKO EBV infected NBLs. (B) Scatterplot displaying DNA and DNA contact changes between WT and LPKO EBV infected NBLs. Red dots are DNA contacts uniquely detected from WT EBV infected NBLs (EBNALP induced contacts), blue dots are DNA contacts uniquely detected from LPKO EBV infected NBLs (EBNALP reduced contacts). Black dots within the green dot line are DNA contacts unchanged between the two groups (Stable contacts). (C) Distribution of DNA contacts from (B) based on DNA distance within the genome. (D) Statistic results of (C).

Appendix Figure S2

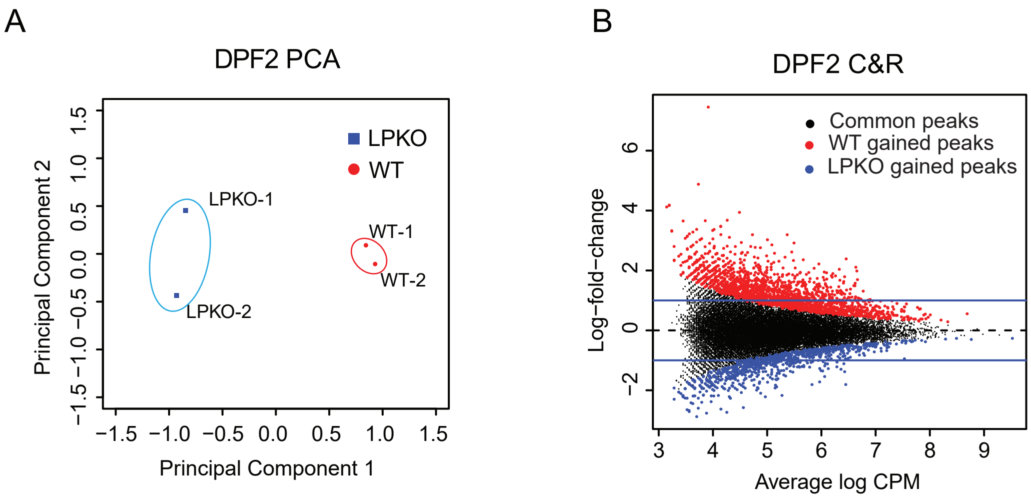

Appendix Figure S2.

(A) PCA analysis of DPF2 CUT&RUN two replicates from WT and LPKO EBV infected NBLs. (B) Scatterplot displaying DPF2 DNA binding loci changes between WT and LPKO EBV infection of NBLs. Red dots are DPF2 CUT&RUN peaks uniquely detected from WT EBV infected NBLs (EBNALP gained peaks), blue dots are DPF2 CUT&RUN peaks uniquely detected from LPKO EBV infected NBLs (EBNALP reduced peaks). Black dots within the green dot line are DPF2 CUT&RUN peaks unchanged between the two groups (Stable peaks).
