# Supplementary figures and images for: Viral oncogene EBNALP regulates YY1 DNA binding and alters host 3D genome organization
Source: EMBO Rep. 2025 Jan 2;26(3):810–35. doi: 10.1038/s44319-024-00357-6 (PMC11811279; doi:10.1038/s44319-024-00357-6)

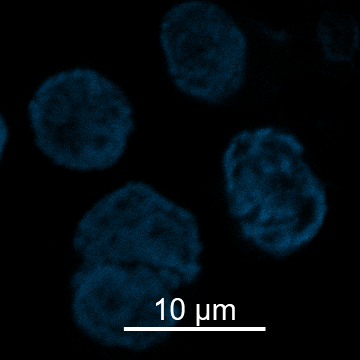

Supplement: Supplementary file 4 — Source data Fig. 2 [file 44319_2024_357_MOESM4_ESM.zip › Figure 2C/DAPI.tif]

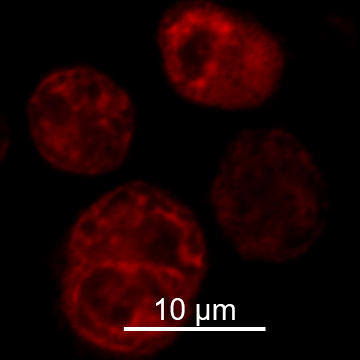

Supplement: Supplementary file 4 — Source data Fig. 2 [file 44319_2024_357_MOESM4_ESM.zip › Figure 2C/DPF2.tif]

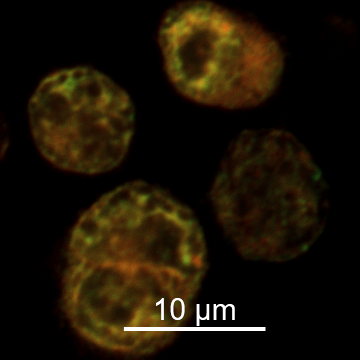

Supplement: Supplementary file 4 — Source data Fig. 2 [file 44319_2024_357_MOESM4_ESM.zip › Figure 2C/EBNALP and DPF2 overlap.tif]

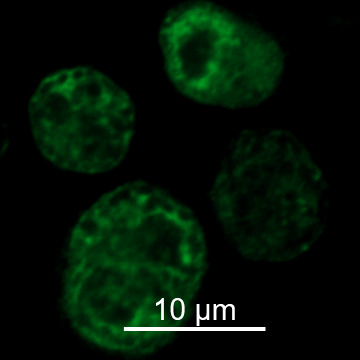

Supplement: Supplementary file 4 — Source data Fig. 2 [file 44319_2024_357_MOESM4_ESM.zip › Figure 2C/EBNALP.tif]

## Slide 1
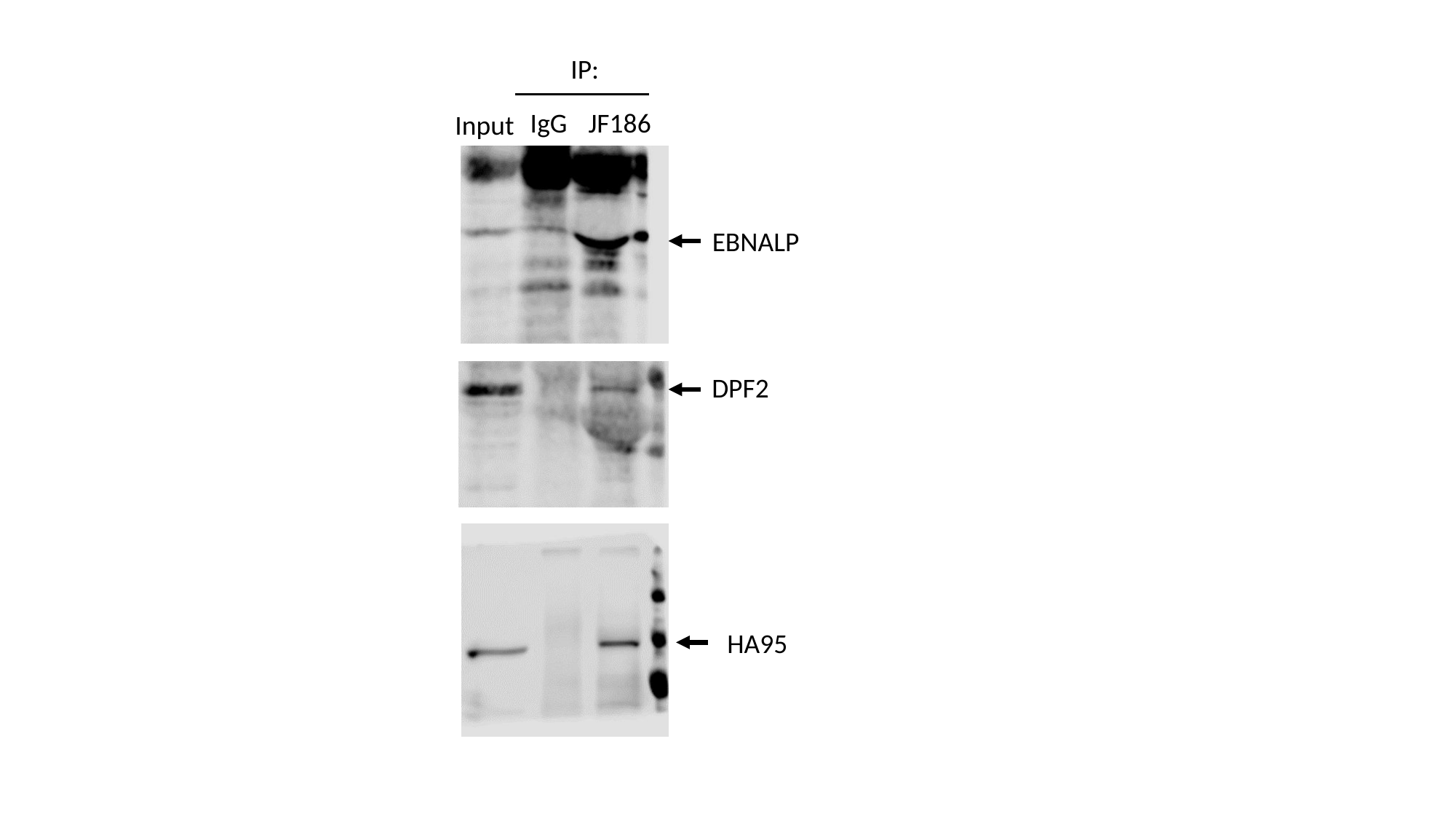

IP:
Input
IgG
JF186
EBNALP
DPF2
HA95

Supplement: Supplementary file 4 — Source data Fig. 2 [file 44319_2024_357_MOESM4_ESM.zip › Figure 2D.pptx]

## Slide 1
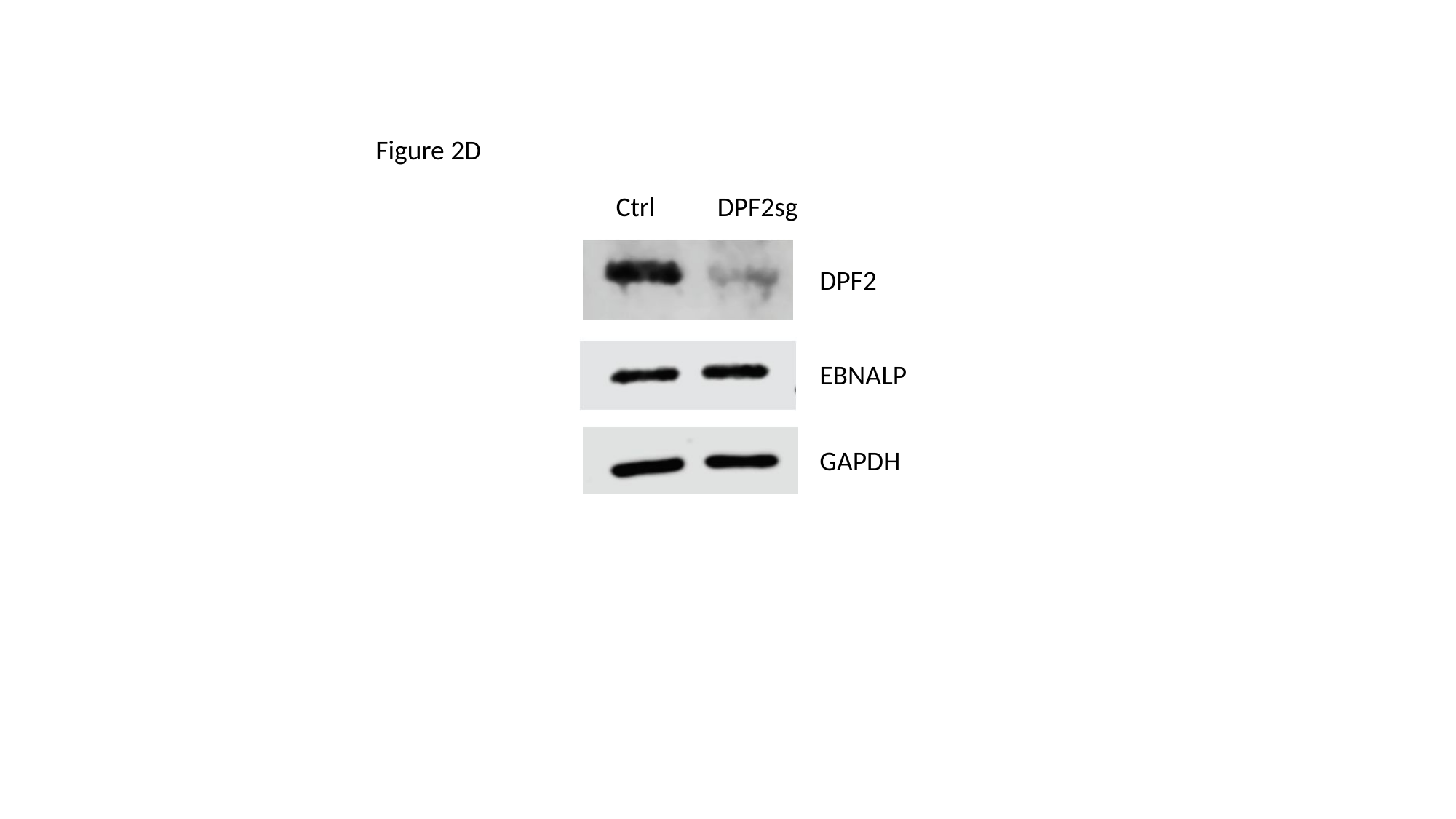

Figure 2D
DPF2sg
Ctrl
DPF2
EBNALP
GAPDH

Supplement: Supplementary file 4 — Source data Fig. 2 [file 44319_2024_357_MOESM4_ESM.zip › Figure 2E_WB.pptx]

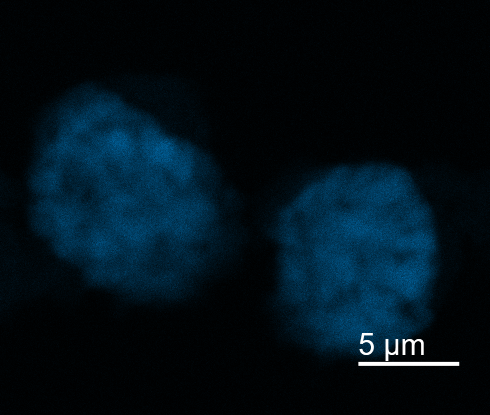

Supplement: Supplementary file 6 — Source data Fig. 4 [file 44319_2024_357_MOESM6_ESM.zip › Figure 4D/DAPI.tif]

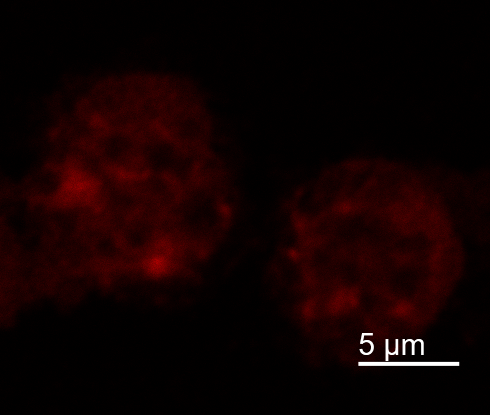

Supplement: Supplementary file 6 — Source data Fig. 4 [file 44319_2024_357_MOESM6_ESM.zip › Figure 4D/EBNALP.tif]

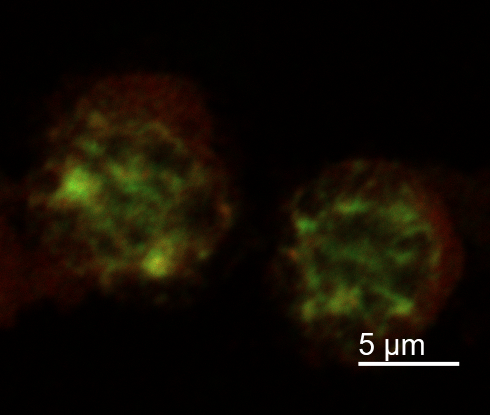

Supplement: Supplementary file 6 — Source data Fig. 4 [file 44319_2024_357_MOESM6_ESM.zip › Figure 4D/YY1 and EBNALP overlap.tif]

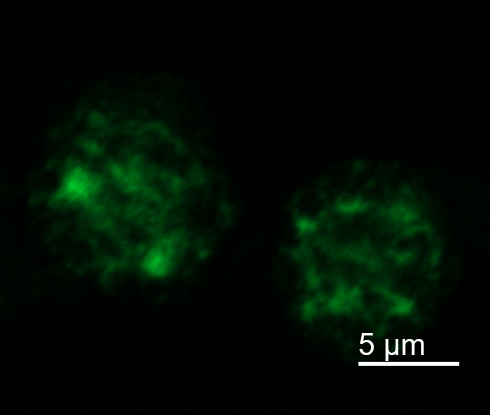

Supplement: Supplementary file 6 — Source data Fig. 4 [file 44319_2024_357_MOESM6_ESM.zip › Figure 4D/YY1.tif]

## Slide 1
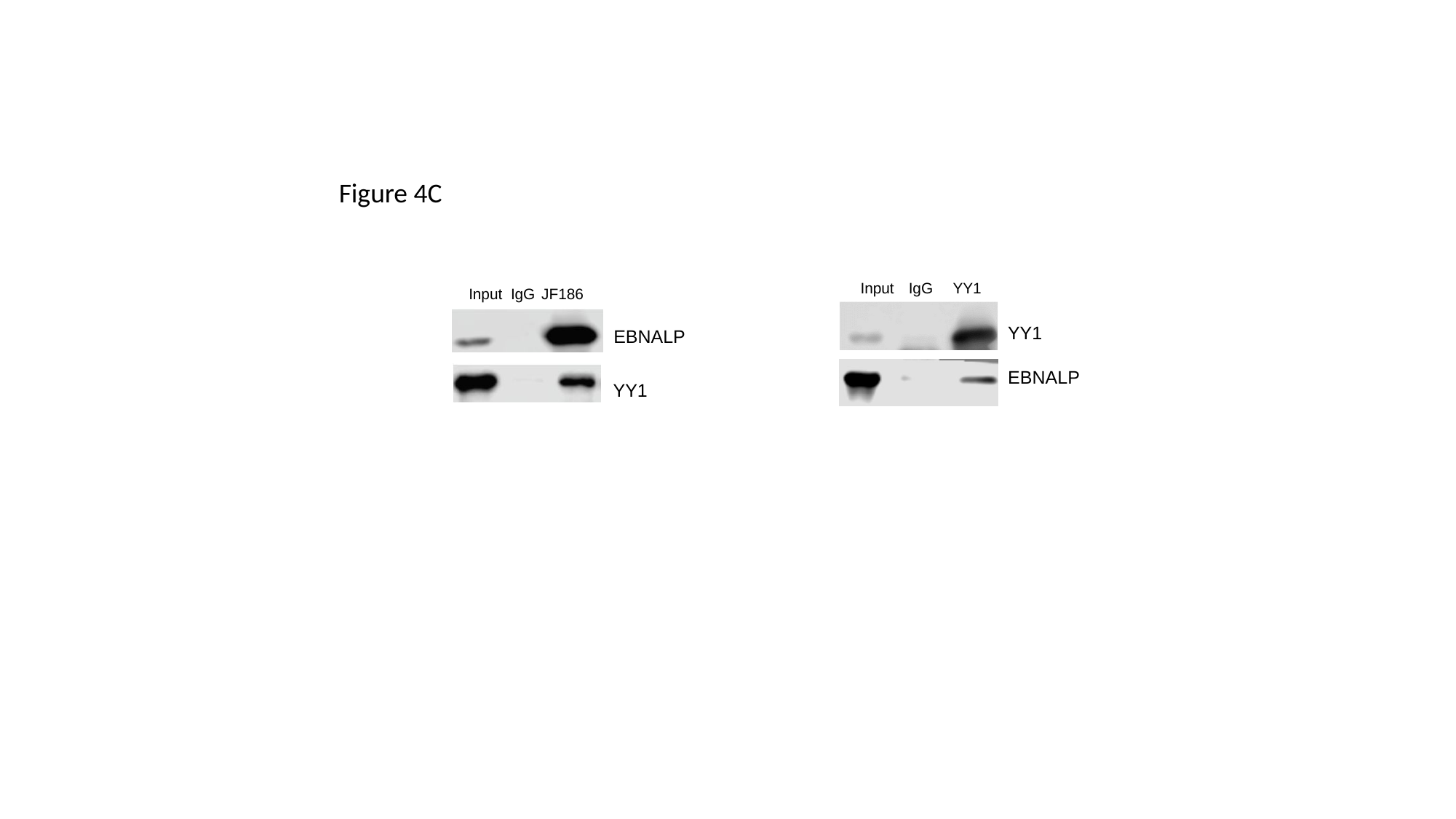

Figure 4C
Input
IgG
YY1
Input
IgG
JF186
YY1
EBNALP
EBNALP
YY1

Supplement: Supplementary file 6 — Source data Fig. 4 [file 44319_2024_357_MOESM6_ESM.zip › Figure 4C.pptx]

## Slide 1
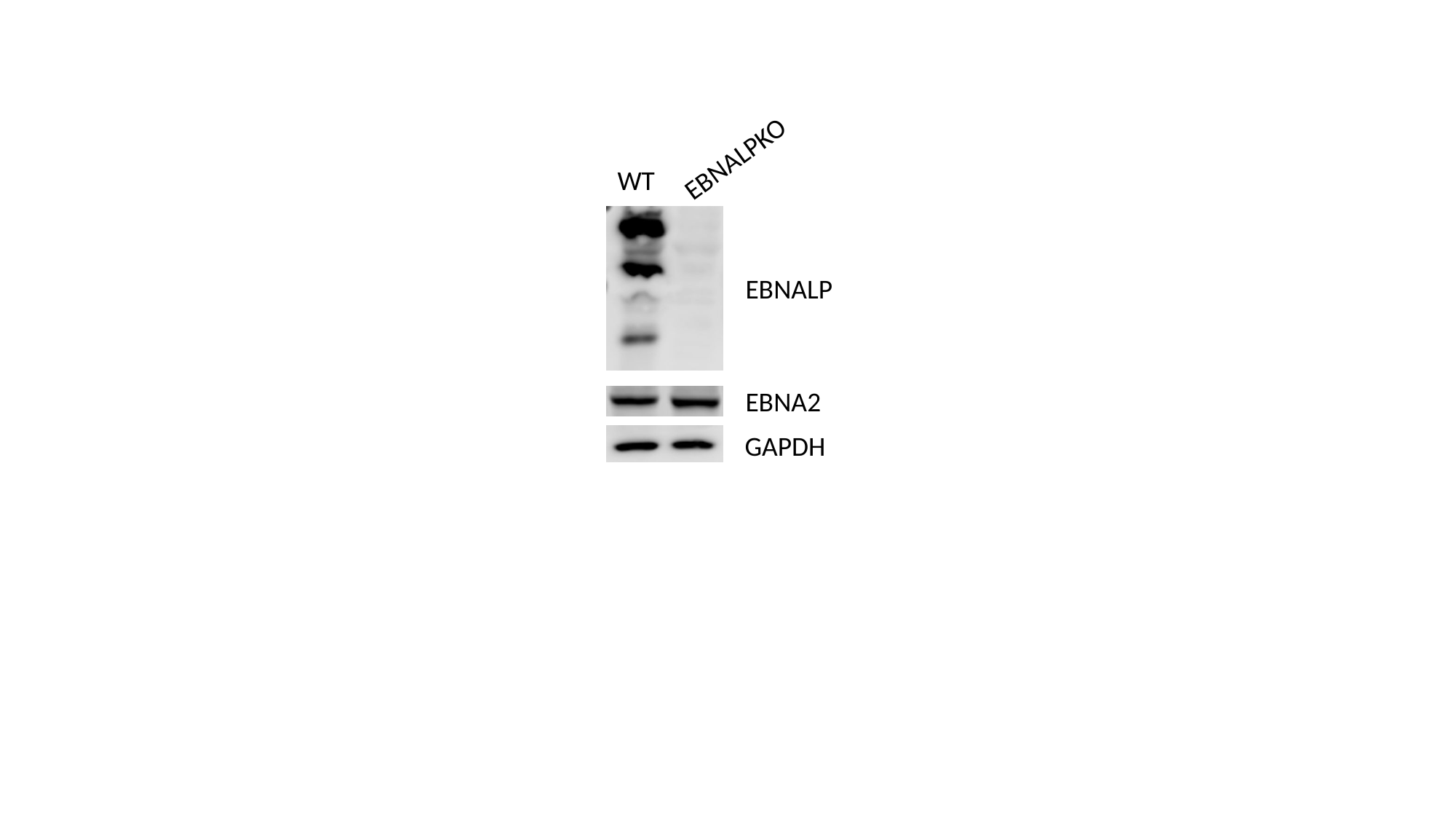

EBNALPKO
WT
EBNALP
EBNA2
GAPDH

Supplement: Supplementary file 7 — EV Figures Source Data [file 44319_2024_357_MOESM7_ESM.zip › EV figure 1/Figure EV1 C.pptx]

## Slide 1
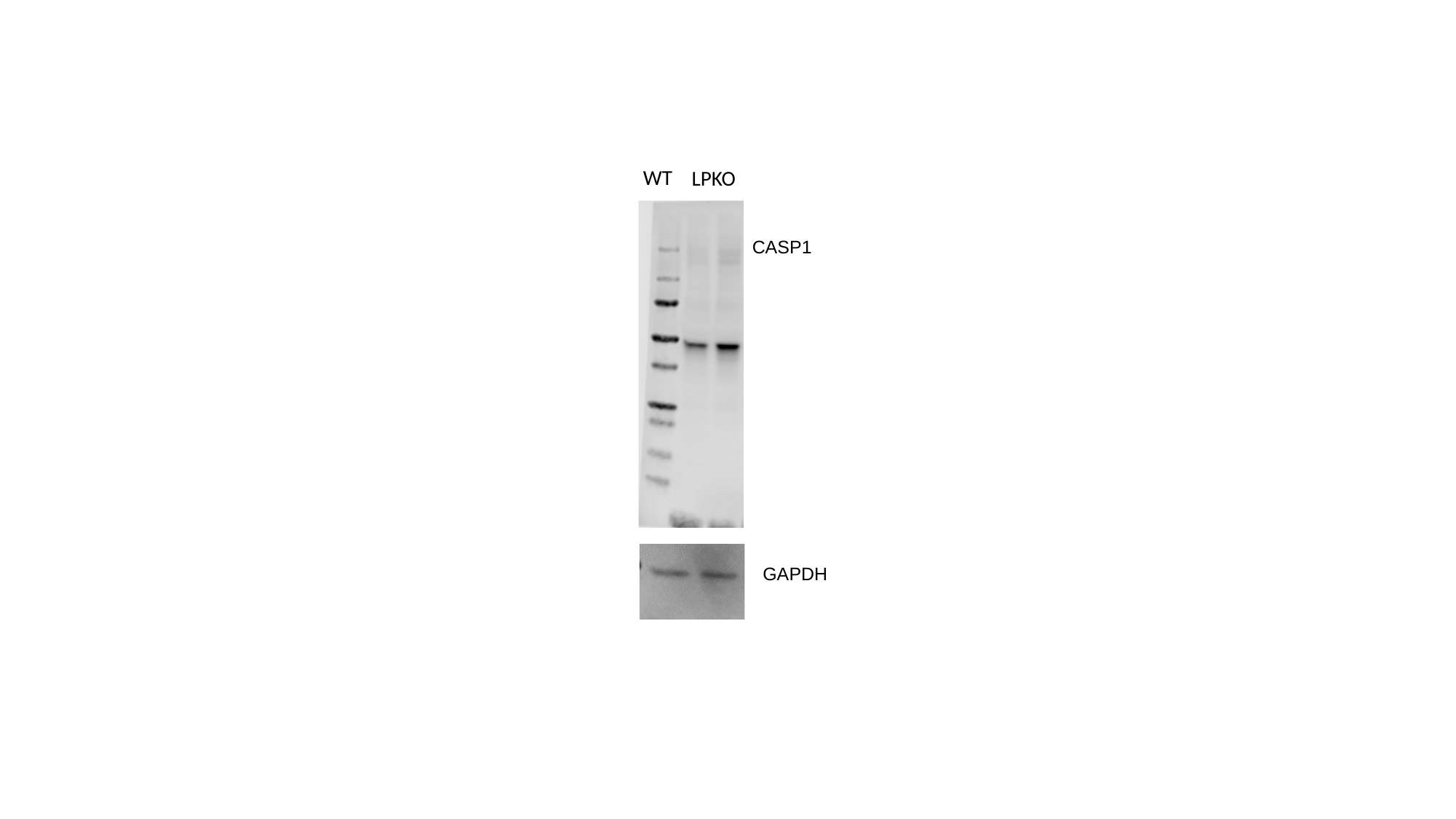

WT
LPKO
CASP1
GAPDH

Supplement: Supplementary file 7 — EV Figures Source Data [file 44319_2024_357_MOESM7_ESM.zip › EV figure 4/Figure EV4 B.pptx]

## Slide 1
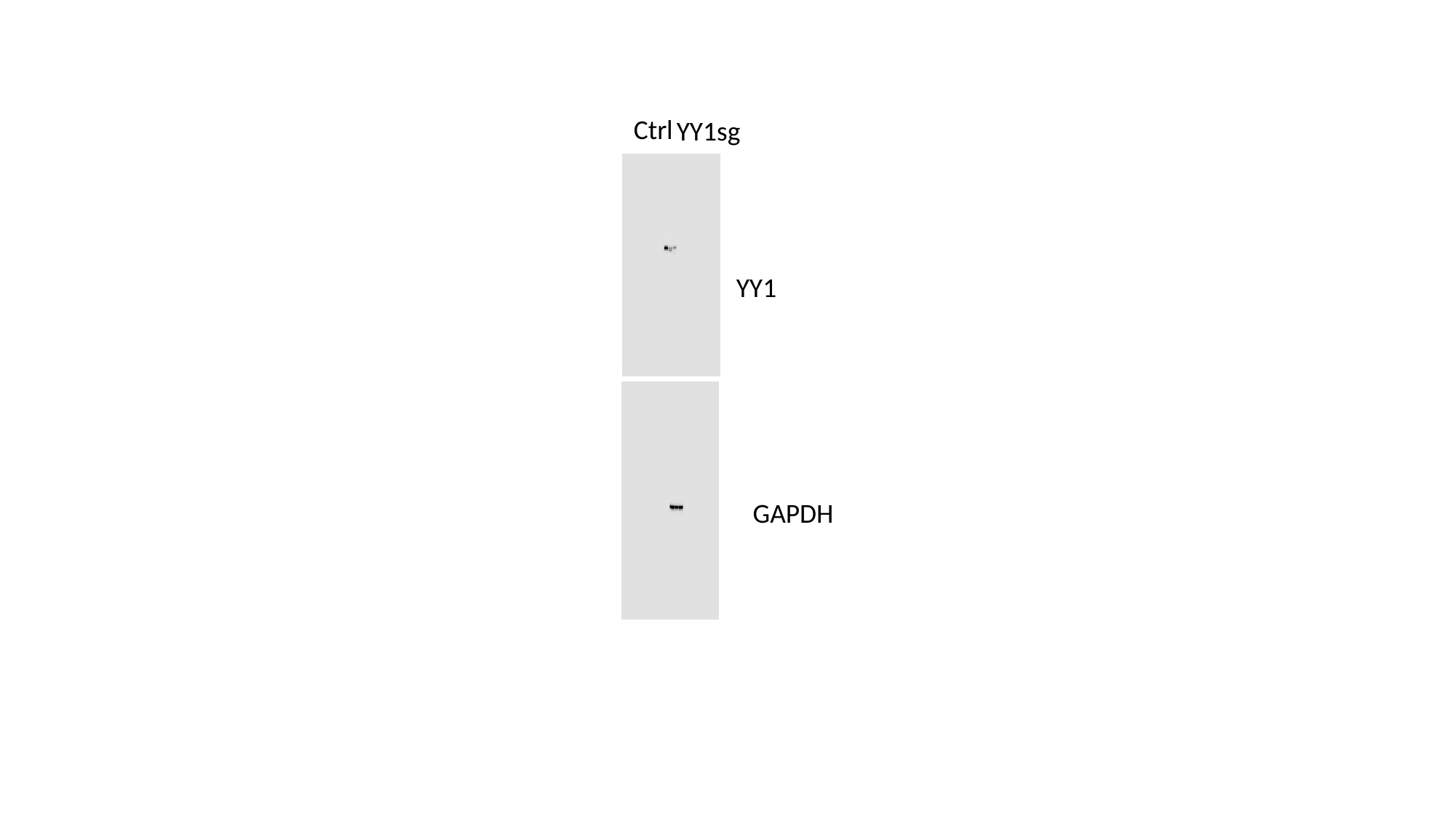

Ctrl
YY1sg
YY1
GAPDH

Supplement: Supplementary file 7 — EV Figures Source Data [file 44319_2024_357_MOESM7_ESM.zip › EV figure 2/Figure EV2 E.pptx]
