# Supplementary material for: Viral oncogene EBNALP regulates YY1 DNA binding and alters host 3D genome organization
Source: EMBO Rep. 2025 Jan 2;26(3):810–35. doi: 10.1038/s44319-024-00357-6 (PMC11811279; doi:10.1038/s44319-024-00357-6)
Supplement: Supplementary file 8 — Expanded View Figures [file 44319_2024_357_MOESM8_ESM.pdf]

Expanded View Figures

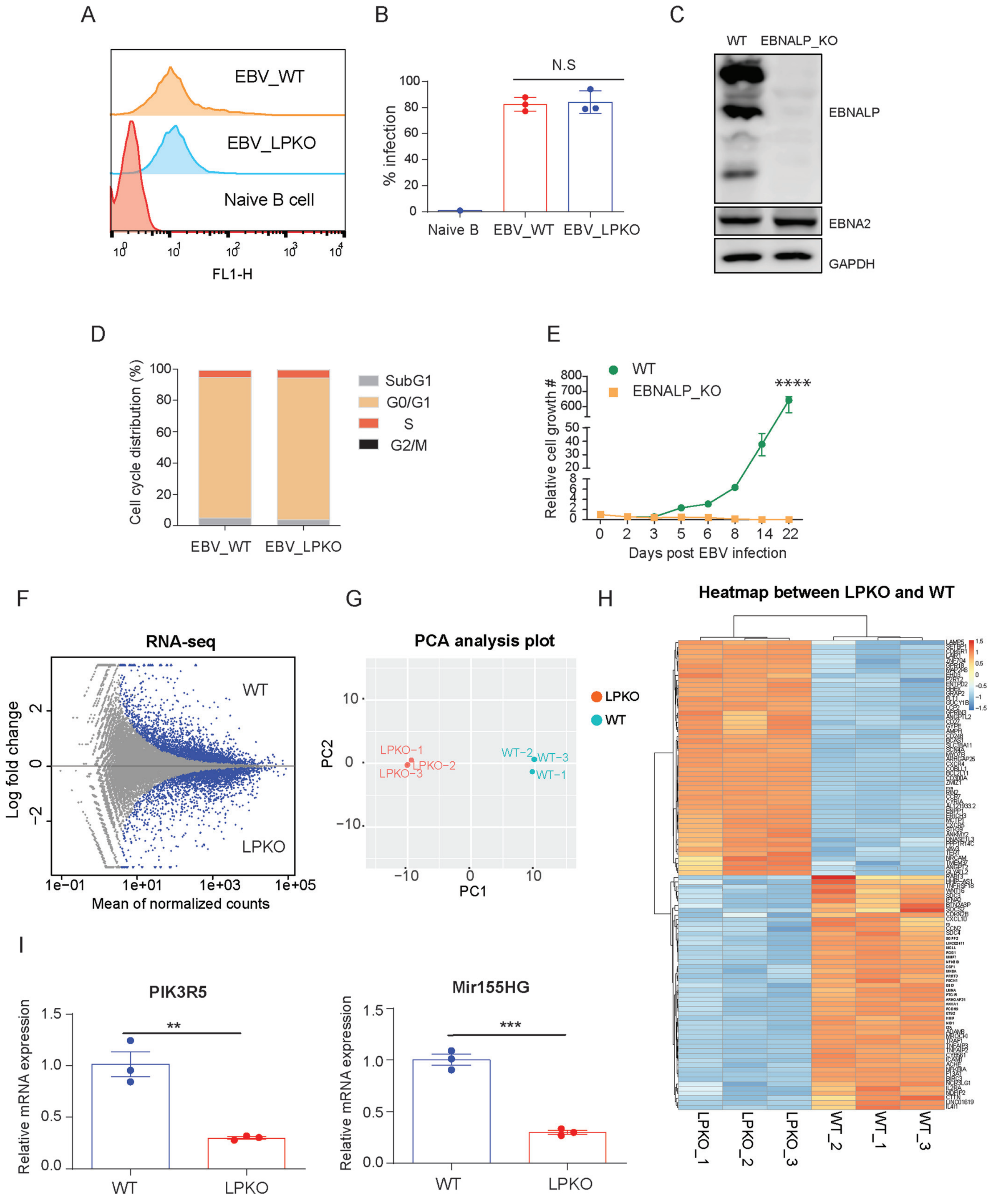

 © The Author(s)

Figure EV1. Characterization of wt and LPKO EBV infected NBLs.

(**A**) Flow cytometry analyses of wt and LPKO EBV-infected NBLs. Uninfected NBLs were used as a negative control. (**B**) Statistic results of percentage of NBLs infected with WT or LPKO EBV ($n = 3$). N.S (no significance). ($n = 3$ biological replicates). (**C**) Western blots detecting EBNALP and EBNA2 expression after WT or LPKO EBV infection of NBLs. GAPDH was used as a loading control. Blots are representative of $n = 2$ replicates. (**D**) Cell cycle analysis of NBLs infected with WT or LPKO EBV. Cell cycle is average from $n = 3$ replicates. (**E**) Relative cell growth curve of NBLs infected with WT or LPKO EBV. Day 0 was normalized to 1. ($n = 6$ biological replicates). Statistical significance was tested between WT and LPKO groups at day 28. (P value < 0.0001 was shown as <0.0001. $P < 0.0001$). (****$P < 0.0001$). (**F**) Scatterplot displaying changes in gene expression (log fold change) against all the genes between WT and LPKO EBV infection of NBLs. Differential genes were shown as blue dots. Genes from WT group were plotted above, and genes from LPKO group were plotted below. (**G**) PCA analysis of RNA-seq three replicates from WT and LPKO EBV-infected NBLs. (**H**) Heatmap displaying some genes that were differentially expressed between WT and LPKO EBV infected NBLs. (**I**) RT-qPCR detecting PIK3R5 and Mir155HG transcription from WT and LPKO infected NBLs. RT-qPCR was performed in $n = 3$ biological replicates. Statistical significance was tested between WT and LPKO groups. (For PIK3R5, $P = 0.004$; MIR155HG, $P = 0.0002$). A two-tailed unpaired t test was used for statistical analyses. The error bars indicate the SEM for the averages of $n = 3$ biological replicates. (**$P < 0.01$, ***$P < 0.001$). Source data are available online for this figure.

© The Author(s)

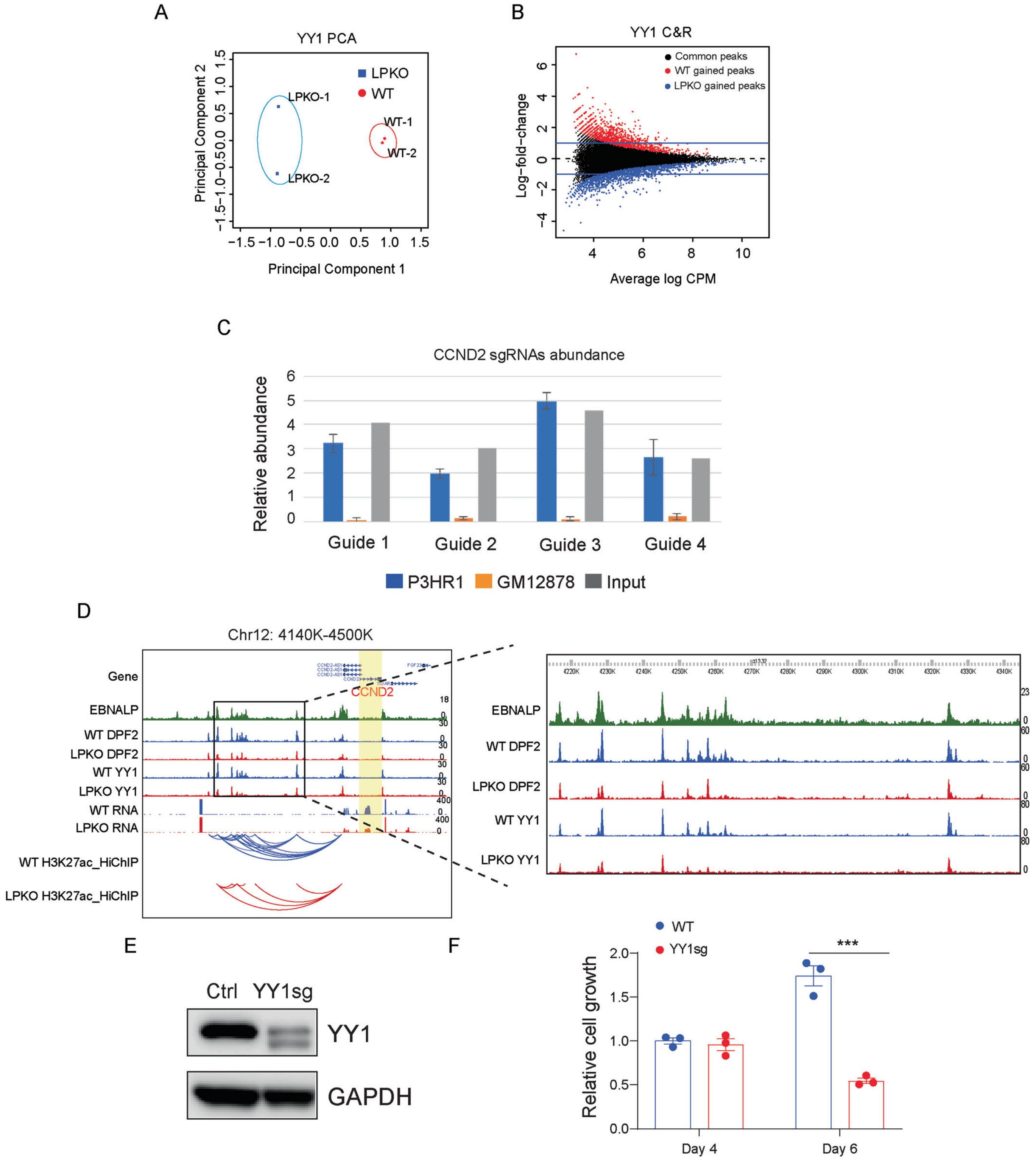

A
YY1 PCA
LPKO
WT
LPKO-1
LPKO-2
WT-1
WT-2
Principal Component 1
Principal Component 2
B
YY1 C&R
Common peaks
WT gained peaks
LPKO gained peaks
Average log CPM
Log-fold-change
C
CCND2 sgRNAs abundance
Relative abundance
Guide 1
Guide 2
Guide 3
Guide 4
P3HR1
GM12878
Input
D
Chr12: 4140K-4500K
Gene
CCND2
EBNALP
WT DPF2
LPKO DPF2
WT YY1
LPKO YY1
WT RNA
LPKO RNA
WT H3K27ac_HiChIP
LPKO H3K27ac_HiChIP
E
Ctrl
YY1sg
YY1
GAPDH
F
WT
YY1sg
***
Relative cell growth
Day 4
Day 6

 © The Author(s)

Figure EV2. Characterization of YY1 DNA binding genome-wide, at CCND2 locus and YY1 depletion.

(**A**) PCA analysis of YY1 CUT&RUN two replicates from WT and LPKO EBV-infected NBLs. (**B**) Scatterplot displaying YY1 DNA-binding loci changes between WT and LPKO EBV infection of NBLs. Red dots are YY1 CUT&RUN peaks uniquely detected from WT EBV-infected NBLs (EBNALP gained peaks), blue dots are YY1 CUT&RUN peaks uniquely detected from LPKO EBV-infected NBLs (EBNALP reduced peaks). Black dots within the green dot line are YY1 CUT&RUN peaks unchanged between the two groups (Stable peaks). (**C**) CCND2 is essential for LCL growth and survival. CCND2 CRISPR depletion prevents LCL growth. CRISPR was from $n = 2$ biological replicates. (**D**) Zoom in on DPF2 and YY1 CUT&RUN tracks from WT and LPKO infected NBLs at CCND2 locus. (**E**) Western blot displaying YY1 protein level after depletion of YY1 with CRISPR-cas9. GAPDH was used as loading control. Blots are representative of $n = 2$ replicates. (**F**) Relative cell growth of WT and YY1 depleted LCLs. Cell growth was monitored by CTG assay. Cell growth is from $n = 3$ replicates. Statistical significance was tested between WT and YY1sg groups. (For Day 6, $P = 0.0006$). A two-tailed unpaired *t* test was used for statistical analyses. The error bars indicate the SEM for the averages. (***$P < 0.001$). Source data are available online for this figure.

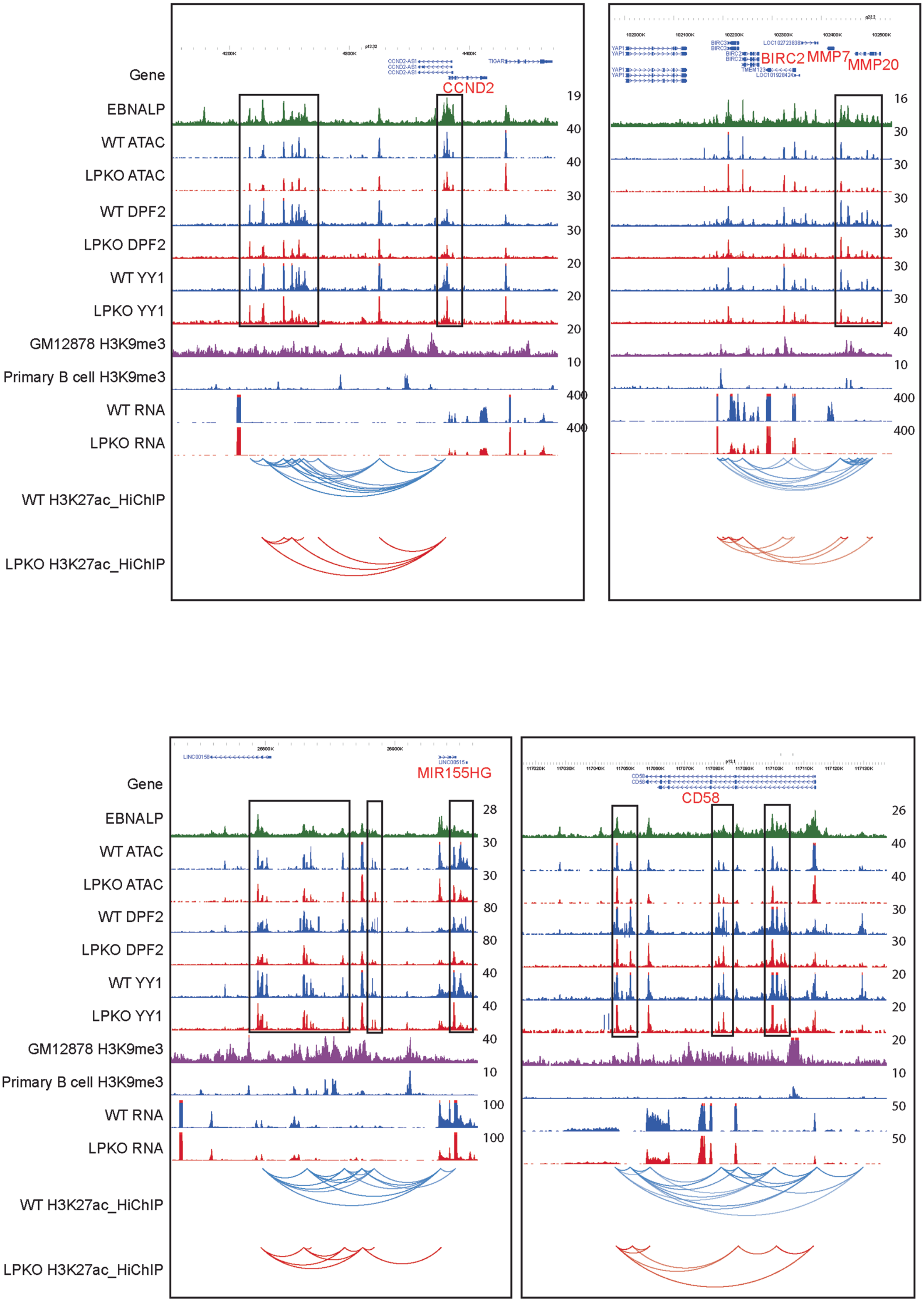
Gene
EBNALP
WT ATAC
LPKO ATAC
WT DPF2
LPKO DPF2
WT YY1
LPKO YY1
GM12878 H3K9me3
Primary B cell H3K9me3
WT RNA
LPKO RNA
WT H3K27ac_HiChIP
LPKO H3K27ac_HiChIP
CCND2
19
40
40
30
30
20
20
20
10
400
400
BIRC2
MMP7
MMP20
16
30
30
30
30
30
30
40
10
400
400
MIR155HG
28
30
30
80
80
40
40
40
10
100
100
CD58
26
40
40
30
30
20
20
20
10
50
50

 © The Author(s)

◀ **Figure EV3. ChIP-seq, Cut&Run, ATAC-seq, RNA-seq, H3K27ac HiChIP tracks from wt or LPKO EBV-infected cells, LCL or RBL H3K9me3 and EBNALP ChIP-seq tracks at the CCND2, BIRC2, MIR155, and CD58 loci are shown.**

Peak height are indicated on the right side of the corresponding tracks.

© The Author(s)

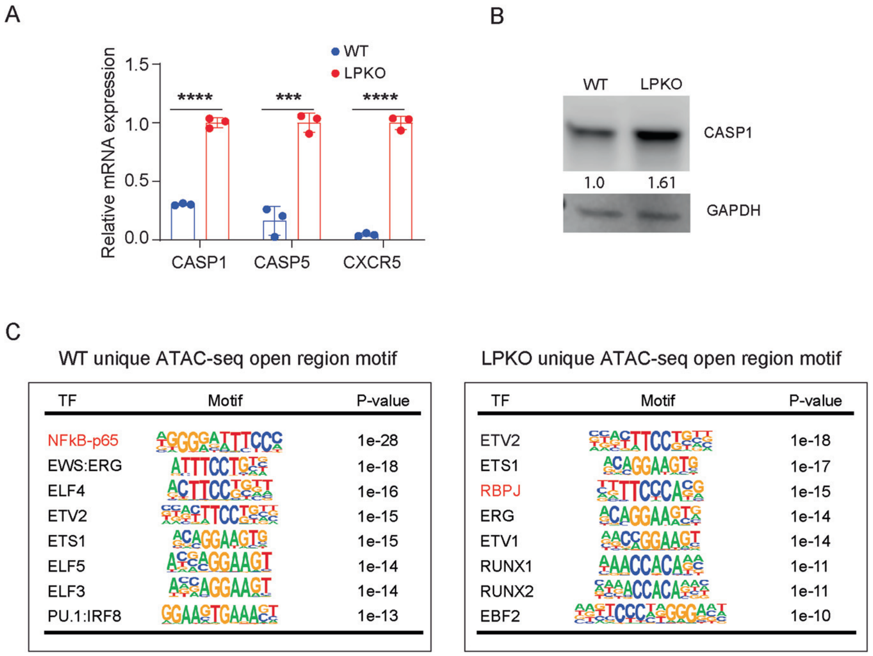

Figure EV4. LPKO EBV induces CASP1 and enrichment of unique motifs at EBNALP regulated ATAC-seq sites.

(**A**) EBNALP downregulates CASP1, CASP5 and CXCR5 transcription. CASP1, CASP5 and CXCR5 mRNA from RNA-seq. Statistical significance was tested between the WT and LPKO groups (*P* value less than 0.0001 is shown as <0.0001. For CASP1, $P < 0.0001$; CASP5, $P = 0.0006$; CXCR5, $P < 0.0001$). (***$P < 0.001$, ****$P < 0.0001$). The error bars indicate the SD for the averages of $n = 3$ biological replicates. (**B**) Western blot detecting CASP1 expression after WT and LPKO EBV infection of NBLs. In the absence of EBNALP, EBV upregulated CASP1 expression, but no cleaved CASP1 protein was detected. GAPDH was used as a loading control. The numbers represent relative protein band intensity measured with Image Studio, quantified by normalizing to GAPDH. Blots are representative of $n = 2$ replicates. (**C**) Motif analysis of EBNALP-induced accessible or compact inaccessible chromatin sites. Source data are available online for this figure.

© The Author(s)
